# Discovering prominent differences in structural and functional connectomes using a multinomial stochastic block model

**DOI:** 10.1162/netn_a_00399

**Published:** 2024-12-10

**Authors:** Nina Braad Iskov, Anders Stevnhoved Olsen, Kristoffer Hougaard Madsen, Morten Mørup

**Affiliations:** Department of Applied Mathematics and Computer Science, Technical University of Denmark, Lyngby, Denmark; Danish Research Centre for Magnetic Resonance, Centre for Functional and Diagnostic Imaging and Research, Copenhagen University Hospital - Amager and Hvidovre, Copenhagen, Denmark

**Keywords:** Structural connectivity, Functional connectivity, Multinomial stochastic block model, Difference modeling, Bayesian inference

## Abstract

Understanding the differences between functional and structural human brain connectivity has been a focus of an extensive amount of neuroscience research. We employ a novel approach using the multinomial stochastic block model (MSBM) to explicitly extract components that characterize prominent differences across graphs. We analyze structural and functional connectomes derived from high-resolution diffusion-weighted MRI and fMRI scans of 250 Human Connectome Project subjects, analyzed at group connectivity level across 50 subjects. The inferred brain partitions revealed consistent, spatially homogeneous clustering patterns across inferred resolutions demonstrating the MSBM’s reliability in identifying brain areas with prominent structure-function differences. Prominent differences in low-resolution brain maps (*K* = {3, 4} clusters) were attributed to weak functional connectivity in the bilateral anterior temporal lobes, while higher resolution results (*K* ≥ 25) revealed stronger interhemispheric functional than structural connectivity. Our findings emphasize significant differences in high-resolution functional and structural connectomes, revealing challenges in extracting meaningful connectivity measurements from both modalities, including tracking fibers through the corpus callosum and attenuated functional connectivity in anterior temporal lobe fMRI data, which we attribute to increased noise levels. The MSBM emerges as a valuable tool for understanding differences across graphs, with potential future applications and avenues beyond the current focus on characterizing modality-specific distinctions in connectomics data.

## Author Summary

We present a probabilistic framework, the multinomial stochastic block model (MSBM), to discover prominent differences between structural and functional high-resolution networks derived from diffusion-weighted MRI and fMRI scans from 250 Human Connectome Project subjects. By varying the number of clusters, the MSBM reveals at different levels of resolution the most prominent differences in how brain regions are functionally and structurally connected. Synthetic experiments and consistent brain map patterns extracted in real high-resolution connectomes highlight the MSBM’s ability to identify brain areas with prominent structure-function differences. This includes prominent differences in the connectivity of the anterior temporal lobes as well as interhemispheric connectivity, which we attribute mainly to the specific technical and methodological limitations of the functional and structural modality, respectively.

## INTRODUCTION

Understanding the organization of the brain is a pivotal pursuit in the field of neuroscience and a fundamental requirement for the potential identification of biomarkers related to system-level neurological disorders. The brain may be viewed as a complex network (connectome) involving two main components: *segregated* neuronal regions and the *integration* connecting these regions (Tononi, Sporns, & Edelman, 1994). The integration of cross-brain functional activity most likely involves signal propagation through an extensive web of axonal anatomical interconnectivity as evidenced by anatomical abundance. However, the precise alignment of anatomical cortico-cortical connections and functional interaction remains unclear.

Conceptualizing the brain as a *graph*—where nodes signify segregated regions and links represent integration—provides a useful framework for modeling its complexity (Bullmore & Sporns, 2009). Two main types of brain graphs are defined through the link measure: Structural connectivity (SC) describes the anatomical organization of the brain through white matter neuronal tracts derived from diffusion-weighted MRI (dMRI), while functional connectivity (FC) refers to the statistical dependence between time series reflecting neural activity, often measured through functional MRI (fMRI). The prevailing assumption in neuroscientific research is that the brain’s functional connections is hinged upon the scaffold of the underlying structural architecture (Babaeeghazvini, Rueda-Delgado, Gooijers, Swinnen, & Daffertshofer, 2021; Hillman, 2014; Huang & Ding, 2016). Studies have, thus , focused on relating the two by, for example, predicting FC from SC (Hinne, Ambrogioni, Janssen, Heskes, & van Gerven, 2014) or revealing underestimated dMRI SC weights from fMRI-established FC (Chu, Parhi, & Lenglet, 2018). Indeed, some studies show that FC partially emerges from SC (Becker et al., 2015; Greicius, Supekar, Menon, & Dougherty, 2009; Sporns, 2014). However, robust functional connections have also been observed between cortical regions lacking strong structural links (Honey et al., 2009; Koch, Norris, & Hund-Georgiadis, 2002; Skudlarski et al., 2010; Vincent et al., 2007).

The most notable difference between structural and functional connectomes is evident in the interhemispherical connections. Tractography-estimated anatomical streamlines are predominantly ipsilateral due to the difficulty of accurately tracking fibers through dense bundles such as the corpus callosum (Crawford, 2022; Jeurissen, Descoteaux, Mori, & Leemans, 2019; Jones, 2010; Škoch et al., 2022), while interhemispheric functional correlations are typically more abundant. Furthermore, the estimation of both connectome types exhibits imperfections—dMRI has notorious difficulties with crossing fiber bundles and probabilistic tractography is constrained by the number of randomly sampled streamlines (typically 5–10 million). Similarly, static FC, measured through interregional Pearson correlation coefficients, is limited by the low sampling rate and relies on the slow blood oxygenation level dependent (BOLD) response such that only slowly evolving (i.e., <0.1 Hz) dynamics may be tracked. A recent study indicated that unihemispheric cortical surface geometry predicts task activity and resting-state FC more parsimoniously than SC (Pang et al., 2023), underscoring the inherent challenge of SC to provide a meaningful lattice for understanding fMRI derived brain activity. Consequently, the direct linkage between SC and FC as measured by contemporary dMRI and fMRI methods remains unclear.

A prominent probabilistic methology for characterizing a graph in terms of segregated units and their integration is the stochastic block model (SBM), which through Bayesian inference quantifies within-graph node similarities using a set of blocks/clusters (segregated regions) with separate intra- and intercluster link densities (integration; Holland, Laskey, & Leinhardt, 1983). This framework has historically been used to elucidate cross-subject FC similarity (Andersen et al., 2014; Baldassano, Beck, & Fei-Fei, 2015; Mørup, Madsen, Dogonowski, Siebner, & Hansen, 2010), SC similarity (Ambrosen, Albers, Dyrby, Schmidt, & Mørup, 2014; Baldassano et al., 2015), and joint similarities both in terms of segregated regions and how they consistently integrate (Andersen et al., 2012) or only in terms of shared segregated regions (Albers et al., 2022). Notably, the SBM is closely related to the widely used modularity optimization procedure for community detection, such that modularity optimization can be considered a special case of maximum likelihood optimization of the degree-corrected SBM (Karrer & Newman, 2011; Newman, 2016). Specifically, modularity assumes a simplified SBM with only two parameters defining the block densities—a single shared within group and a single shared between group parameter. Importantly, the SBM reduced to such two-parameter block model provides a procedure to automatically learn these two parameters, which corresponds to tuning the resolution used in the generalized modularity optimization framework of Reichardt and Bornholdt (2006). See also Newman (2016) for details.

Examining the prominent *differences* between the two modalities may offer a more insightful perspective rather than focusing on similarities. The aim of this article is to provide a methodology to efficiently uncover these prominent and systematic differences between SC and resting-state FC at varying scales of the connectome. To achieve this, we explore a generalization of the SBM called the multinomial SBM (MSBM), which replaces the traditional within-graph Bernoulli or Poisson likelihood (Schmidt & Morup, 2013) with an across-graphs multinomial likelihood (Mørup, Glückstad, Herlau, & Schmidt, 2014). Specifically, we develop a parametric as opposed to nonparametric (Mørup et al., 2014) formulation of the MSBM that allows to explicitly control the number of clusters and, thereby, the “resolution” of the inferred segregated structure. We presently demonstrate how the MSBM explicitly characterizes prominent connectivity differences across a set of graphs, for example, structural and functional connectomes. Node-specific differences could be obtained through simple subtraction of graphs. However, at high resolution, differences can easily be driven by noisy connectivity estimates. Formulating the data within a probabilistic framework allows us to learn and analyze meaningful and prominent components to reveal systematic patterns related to SC and FC differences.

To illuminate the merits and limitations of the MSBM in uncovering differences across a set of graphs, we first systematically conduct a series of tests on synthetic connectomes. Subsequently, using high-resolution (59,412 nodes) structural and functional cortical connectomes from 250 Human Connectome Project (HCP) subjects (Van Essen et al., 2012), we infer the prominent differences between the two types of connectomes while accounting for within-modality differences considering simultaneously five graphs from each modality defined as the aggregated connectivity of 50 nonoverlapping subjects. We hypothesize that for a small number of clusters, the MSBM will predominantly unveil low structural and high functional interhemispheric connectivity. However, for a larger number of clusters, we expect that the MSBM can provide a detailed characterization of prominent and potentially unknown SC and FC differences that can occur both intra- and interhemispherically. The approach can thereby provide a detailed map of the most prominently *segregated regions* that systematically differ in terms of their structural and functional *integration*, highlighting the central differences of high-resolution functional and structural connectomes derived from fMRI and dMRI.

## MATERIALS AND METHODS

The SBM is a parametric generative model used for identifying communities (i.e., clusters) in graphs (Holland et al., 1983). In the Bayesian framework, important model parameters are defined as random variables using probability distributions reflecting their uncertainty. This includes specifying a likelihood function, imposing priors for latent parameters, and subsequently inferring their optimal values using, for example, sampling methods through Bayes’ rule.

**Figure 1.**
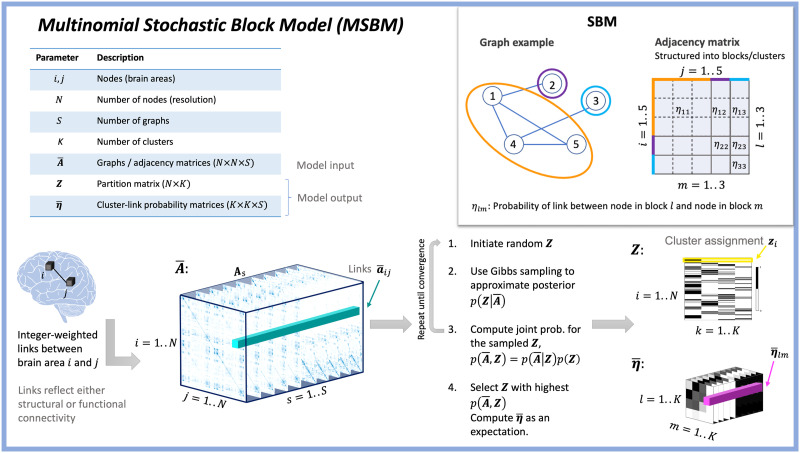
Visualization of the MSBM framework. Each graph adjacency matrix **A**_*s*_ contains integer-weighted links between nodes *i* and *j*. The three-dimensional array $\bar{A}$ is constructed by stacking all graphs. Using an initial partition, sufficient statistics are computed and subsequently used to estimate the MSBM likelihood considering an across-graph comparative cluster link probability vector *η*_*lm*_ constrained to sum to 1 across the graphs (i.e., ∑_*s*_ *η*_*lms*_ = 1). Using Gibbs sampling, the partition matrix **Z** (which is a matricization of the partition vector **z**) is inferred and the joint probability of the sampled solution is computed. This process repeats for a specified number of iterations or until convergence. We finally report the MAP partition having the highest joint distribution across the sampling procedure. The model uses a multinomial likelihood for the link probability to allow for modeling of both binary and integer-weighted graphs across the shared partition matrix **Z**. The MSBM thereby identifies clusters that exhibit prominent connectivity differences across the considered graphs.

The traditional SBM partitions a single, binary graph **A** into a set of *K* blocks/clusters in which nodes assigned to the same cluster *l* are assumed to have the same probability of linking to the nodes within the cluster and to nodes in other clusters *m* as specified by the within- and between-cluster link probability matrix ***η*** with elements *η*_*lm*_ (see also Figure 1 inset). Thus, the learned parameters are the partition vector **z** = [*z*_*i*_, …, *z*_*N*_] across *N* nodes and the symmetric cluster link probability matrix ***η*** such that within-cluster link probabilities are specified along the diagonal and between-cluster link probabilities in the off-diagonal elements. Multiple extensions to this framework have been proposed, notably including extending the modeling dimension along a third dimension, allowing for modeling a set of *S* graphs, for example, assuming both the partitions and the link probability matrix ***η*** are shared across graphs (Andersen et al., 2012) or assuming shared partitions but graph-specific probability matrices such that $\bar{\eta }$ is a third-order array with elements *η*_*lms*_ (Albers et al., 2022; Mørup et al., 2010).

Our proposed model is an extension of the model originally proposed in Mørup et al. (2014) in the context of structuring binary similarity matches, which assumes a multinomial likelihood across the third dimension with an associated probability distribution specifying the tendency to observe links for each graph (Figure 1). Thereby, the cluster link probability matrix $\bar{\eta }$ forms a three-way array with the constraint that the elements are nonnegative (*η*_*lms*_ ≥ 0) and sum to one along the third mode ∑_*s*_ *η*_*lms*_ = 1 ∀ *l*, *m*. Clusters are estimated across a collection of graphs $\bar{A}$, representing, for example, subjects, domains, or modalities. The graph-specific link probabilities ***η***_*s*_ allow for analyzing general trends and variations across the set of graphs, and the sum-to-one constraint requires the allocation of high values in ***η***_*s*_ to be compensated by comparatively lower values for other graphs. Crucially, as we will presently demonstrate, the MSBM optimizes for the detection of differences across $\bar{A}$.

The generative process of the MSBM is given by

$$z∼\text{DirMult}\left(\alpha_{0}/K\cdot 1_{K}\right),\;\text{Partition}\;\text{of}\;\text{nodes}\;\text{into}\;K\;\text{clusters},$$ (1)

$${\bar{\eta }}_{l,m}∼\text{Dir}\left({\bar{\eta }}_{0}\right),\;\text{Probability}\;\text{of}\;\text{link}\;\text{between}\;\text{cluster}‐\text{pair}\;\left(l,m\right)\;\text{across}\;\text{graphs},$$ (2)

$${\bar{a}}_{i,j}∼\text{Mult}\left({\bar{\eta }}_{z_{i},z_{j}}\right),\;\text{Link}\;\text{between}\;\text{node}\;\text{pairs}\;\left(i,j\right)\;\text{across}\;\text{graphs}.$$ (3)

where **z** = [*z*_*i*_, …, *z*_*N*_] denotes the cluster assignment vector across nodes, *α*_0_ is the concentration parameter specifying how heterogeneous the size of the partitions should be, $\bar{\eta }$_*l*,*m*_ denotes the cluster link probability vector (tube) across graphs for cluster pair *l*, *m*, whereas $\bar{\eta }$_0_ denotes the a priori assumed concentration parameter vector across graphs. Finally, $\bar{a}$_*i*,*j*_ denotes the observed link (count) vector across graphs for node pair *i*, *j*. Notably, the multinomial (Mult) distribution allows for not only binary but also integer-weighted observed adjacency matrices. The Dirichlet (Dir) distribution is here imposed as a prior on the cluster link probabilities as it directly imposes the sum-to-one constraint of $\bar{\eta }$_*l*,*m*_ and due to its mathematical convenience serving as conjugate prior to the multinomial distribution allowing for analytical marginalization forming the DirMult distribution. By marginalizing $\bar{\eta }$_*l*,*m*_, the joint distribution is given by:

$$p\left(\bar{A},z|{\bar{\eta }}_{0},\alpha \right)=\int p\left(\bar{A},z,\bar{\eta }|{\bar{\eta }}_{0},\alpha \right)d\bar{\eta }=\underset{P\left(\bar{A}|z,\eta_{0}\right)}{\underbrace{C\cdot \prod_{l\geq m}^{K}\frac{B\left({\bar{\nu }}_{l,m}+{\bar{\eta }}_{0}\right)}{B\left({\bar{\eta }}_{0}\right)}}}\cdot \underset{P\left(z|\alpha_{0}\right)}{\underbrace{\frac{B\left(n+\alpha_{0}/K\cdot 1_{K}\right)}{B\left(\alpha_{0}/K\cdot 1_{K}\right)}}}$$ (4)

which can be recognized as the product of two DirMult distributions. *B* here denotes the generalized beta function, that is, *B*(***x***) = $\frac{\prod_{t}\Gamma \left(x_{t}\right)}{\Gamma \left(\sum_{t}x_{t}\right)}$, and *C* = ∏_*i*>*j*_ *B*($\bar{a}$_*ij*_)^−1^ denotes a term constant wrt. the model parameters. $\bar{\nu }$_*l*,*m*_ denotes the vector containing the number of links between cluster pair *l*, *m* across graphs (sufficient statistics), that is, *ν*_*l*,*m*,*s*_ ≡ ∑_*z*_*i*_∈*l*,*z*_*j*_∈*m*_ *a*_*i*,*j*,*s*_, whereas *n*_*l*_ = $\sum_{i=1}^{N}$ 𝟙_*l*_(*z*_*i*_) denotes the number of *z*_*i*_s with value *l* (number of nodes in cluster *l*) and **n** = [*n*_1_, …, *n*_*L*_] denotes the vector with elements *n*_*l*_. Details of the derivation of Equation 4 is provided in the Supporting Information. In the following, we will, for brevity, ignore the above distribution’s dependency on $\bar{\eta }$_0_ and *α*_0_.

Unfortunately, the inference of ***z*** is intractable and we therefore need to infer the posterior using approximate inference methods. Specifically, as in Mørup et al. (2014), we use a Markov chain Monte Carlo method known as Gibbs sampling (Geman & Geman, 1984). Gibbs sampling is used to approximate multivariate distributions, and it can be seen as a special case of Metropolis–Hastings (MH) sampling, where the proposal is always accepted (Bishop, 2006), whereas MH sampling can suffer from low acceptance ratio. In Gibbs sampling, we iteratively sample the variables (in this case each cluster assignment *z*_*i*_) from their conditional distribution *p*(*z*_*i*_ = *l*∣$\bar{A}$, **z**^\*i*^), where *z*_*i*_ denotes the cluster assignment of node *i* and **z**^\*i*^ denotes the partition vector for all the cluster assignments except *z*_*i*_. Note that we compute the probability of each node *i* belonging to each cluster *l* ∈ {1 … *K*} given that the previous cluster assignment for node *i* is not included.

$$\underset{\text{Conditional}\;\text{posterior}}{\underbrace{P\left(z_{i}=l|\bar{A},z^{\backslash i}\right)}}=\frac{P\left(\bar{A},z_{i}=l|z^{\backslash i}\right)}{\sum_{m}P\left(\bar{A},z_{i}=m,z^{\backslash i}\right)}$$ (5)

where the conditional joint probability is given by

$$P\left(\bar{A},z_{i}=l|z^{\backslash i}\right)=\underset{\text{Conditional}\;\text{likelihood}}{\underbrace{p\left(\bar{A}|z_{i}=l,z^{\backslash i}\right)}}\underset{\text{Conditional}\;\text{prior}}{\underbrace{p\left(z_{i}=l|z^{\backslash i}\right)}}\propto \frac{\prod_{m}^{K}B\left({\bar{\nu }}_{l,m}+{\bar{\eta }}_{0}\right)}{\prod_{m}^{K}B\left({\bar{\nu }}_{l,m}^{\backslash i}+{\bar{\eta }}_{0}\right)}\left(n_{l}^{\backslash i}+\alpha_{0}\right)$$ (6)

The derivation of the conditional posterior for the parametric MSBM including the derivation of the respective conditional likelihood *P*($\bar{A}$∣*z*_*i*_ = *l*, **z**^\*i*^) and conditional prior *P*(*z*_*i*_ = *l*∣**z**^\*i*^) is provided in the Supporting Information. We further infer ***η***_0_ and *α*_0_ using a MH procedure as suggested in Mørup et al. (2014) in which we impose an improper uniform prior, transform the nonnegative variables to the unconstrained domain using log-transformation, and use a zero-mean normal distribution as proposal distribution with *σ* = 0.1. During each iteration, we ran a full Gibbs sweep of all the elements of ***z***, 100 MH steps of *α*_0_, and 10 MH steps of each element in ***η***_0_.

After inferring the partition vector **z** and sampling the hyperparameters *α* and $\bar{\eta }$_0_, the cluster link probabilities between cluster pairs *l*, *m* for each graph *s* are computed as an expectation of the Dirichlet distribution with concentration parameter $\bar{\nu }$_*l*,*m*_ + $\bar{\eta }$_0_ (expression is given in Supporting Information Equation S9), that is,

$$𝔼\left[\eta_{l,m,s}\right]=\frac{\nu_{l,m,s}+\eta_{0,s}}{\sum_{s=1}^{S}\nu_{l,m,s}+\eta_{0,s}}$$ (7)

where *ν*_*l*,*m*,*s*_ is the number of links between cluster *l*, *m* for graph *s*. These are the elements of ***ν***_*s*_ and can be computed using

$$\nu_{s}={ZA}_{s}Z^{T},$$ (8)

where **Z** is the binary partition matrix of size (*N* × *K*) computed by listing each unique element of **z** in the respective row and column of **Z**.

Since the MSBM is a probabilistic model that optimizes for the most prominent differences across graphs, a low cluster link probability for one graph will lead to higher cluster link probability for the other. In order to account for this when visualizing the results, the entropy of the cluster link probabilities across the multiple graphs is considered, which explicitly assigns high entropy where differences are prominent in terms of link counts, weighing differences by their importance. The entropy for the Dirichlet distribution is given by

$$\begin{array}{l} H\left({\bar{\nu }}_{l,m}+{\bar{\eta }}_{0}\right)=\log B\left({\bar{\nu }}_{l,m}+{\bar{\eta }}_{0}\right)+\left(\left(\sum_{s=1}^{S}\nu_{l,m,s}+\eta_{0,s}\right)-S\right)\psi \left(\sum_{s=1}^{S}\nu_{l,m,s}+\eta_{0,s}\right)\\ \;-\sum_{s=1}^{S}\left(\nu_{l,m,s}+\eta_{0,s}-1\right)\psi \left(\nu_{l,m,s}+\eta_{0,s}\right) \end{array}$$ (9)

where *ψ*(*a*) = $\frac{\Gamma^{\prime }\left(a\right)}{\Gamma \left(a\right)}$ is the digamma function.

We note that the number of clusters, *K*, functions as a *maximum* number of clusters. During inference, the model may remove clusters if these are not deemed significant. Thus, in our experiments, *K* will denote the initial number of clusters. However, in our experience, given sufficiently complex data such as neuroimaging data, the number of clusters will not decrease from the initial value.

Code for MSBM is available at https://github.com/Ninaiskov/ConnDiff-MultSBM. For detailed derivations of the MSBM, we defer the reader to the Supporting Information.

### Normalized Mutual Information

In order to evaluate the similarity between two partitions **z** and **z**′, we used normalized mutual information (NMI) as the evaluation metric. For *l* = 1, …, *K* clusters, the NMI is defined by

$$\begin{array}{l} \text{NMI}\left(z^{\prime },z\right)=\frac{2\text{MI}\left(z^{\prime },z\right)}{\text{MI}\left(z^{\prime },z^{\prime }\right)+\text{MI}\left(z,z\right)}\\ \text{MI}\left(z^{\prime },z\right)=\sum_{\text{l}\prime ,\text{l}}\text{p}\left({\text{l}}^{\prime },\text{l}\right)\log\frac{\text{p}\left({\text{l}}^{\prime },\text{l}\right)}{\text{p}\left({\text{l}}^{\prime }\right)\text{p}\left(\text{l}\right)}\\ \text{p}\left({\text{l}}^{\prime },{\text{l}}^{\prime }\right)=\frac{1}{\text{N}}\sum_{\text{i}}\delta \left(z_{\text{i}},l^{\prime }\right)\delta \left(z_{\text{i}},l\right) \end{array}$$ (10)

where *δ*(*a*, *b*) = 1 if *a* = *b*, and 0 otherwise. The NMI provides a score between 0 and 1 that is invariant to permutations of components (Olsen, Høegh, Hinrich, Madsen, & Morup, 2022). We define models achieving a pairwise NMI below 0.6 as unstable, models with a pairwise NMI between 0.6 and 0.8 as semistable, and models with a pairwise NMI above 0.8 as stable.

### Adjusted Mutual Information

When comparing partitions with differing number of clusters, it is advantageous to compute the adjusted mutual information, which corrects for the expected mutual information between parcellations of specific sizes:

$$\text{AMI}\left(z^{\prime },z\right)=\frac{\text{MI}\left(z^{\prime },z\right)-{𝔼}_{P}\left[\text{MI}\left(Pz^{\prime },z\right)\right]}{\frac{\text{MI}\left(z^{\prime },z^{\prime }\right)+\text{MI}\left(z,z\right)}{2}-{𝔼}_{P}\left[\text{MI}\left(Pz^{\prime },z\right)\right]}$$ (11)

Here, we computed the expectation over all possible permutations **P** through Monte Carlo sampling of random permutation matrices given for sample *s* by **P**^(*s*)^ and given the random permuted clustering **P**^(**s**)^**z**′, which was compared to **z** and averaged across 1,000 sampling iterations.

### Data

#### Synthetic data.

**Figure 2.**
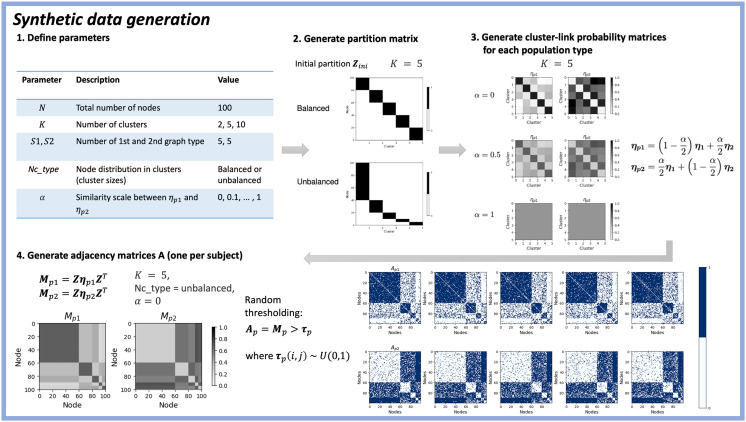
Synthetic data generation process. (1) Relevant parameters are given in the table. (2) We generated a synthetic binary partition matrix **Z** of size *N* × *K*, with elements indicating cluster assignments for each node. To test the effect of different cluster sizes, we used the parameter Nc_type to vary between a balanced and unbalanced number of nodes for each cluster. If Nc_type is unbalanced, then we used predefined uneven cluster sizes given in Supporting Information Table S1. An overview of the initial partitions of nodes into clusters are given in Supporting Information Table S1. (3) We let *p*1 and *p*2 denote the two different populations of graphs. In order to systematically vary the difference between ***η***_*p*1_ and ***η***_*p*2_, we used a similarity scale *α*. ***η***_**1**_ and ***η***_**2**_ are defined as *K* × *K* matrices having high and low within-cluster link probabilities, respectively. An overview of the graph-type-specific cluster link probability matrices for the two populations are given in Supporting Information Table S3. (4) Finally, adjacency matrices were created for each population by extracting all relevant cluster link probabilities for each cluster pair. This results in one matrix **M** per considered graph population (i.e., *p*1 or *p*2) with shape *N* × *N*. For each realization of a graph from a given population, we thresholded **M** using uniformly generated random values for each matrix entry and each graph. In order to make each adjacency matrix undirected (symmetric), we defined **A** as the sum of the upper triangular part of the matrix and the transposed of the upper triangular part of the matrix. We also excluded the main diagonal (all entries in the main diagonal will be 0, i.e., no self-linking nodes). Finally, the adjacency matrices were concatenated to an array of size *N* × *N* × (*S*1 + *S*2).

To test the properties of the MSBM under controlled conditions, we constructed a synthetic dataset mimicking a population of two different groups (e.g., SC and FC graphs). Specifically, the dataset is a set of graphs, where each graph has a group-specific connectivity pattern (cluster interactions) defined by the cluster link probability matrices, ***η***_*p*1_ and ***η***_*p*2_, where *p*1 and *p*2 denote Population 1 and 2. The generation process is divided into four overall steps, which are visualized in Figure 2.

The cluster link probability matrices were constructed such that when *α* = 0 then ***η***_*p*1_ = ***η***_1_ and ***η***_*p*2_ = ***η***_2_, forming two different connectivity structures, whereas when *α* = 1, the connectivity structures are identical: ***η***_*p*1_ = ***η***_*p*1_ = $\frac{1}{2}$***η***_1_ + $\frac{1}{2}$***η***_2_. Using the initial partitions (Supporting Information Table S2) and cluster link probability matrices (Supporting Information Table S3), we constructed the partitions expected to be found by the model. Since the model looks for clusters with *varying* cluster link probability across graphs, we would expect the model to find all *K* clusters if the population cluster link probability matrices are completely different. If they are the same, we would expect the model to find only one cluster, reflecting no difference observed in connectivity structure across the graphs. The expected partitions for Nc_type = unbalanced is seen in Supporting Information Table S4.

#### HCP data.

**Figure 3.**
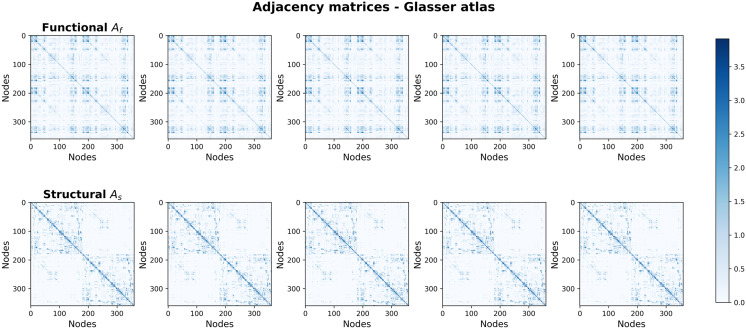
Input connectomes visualized in the Glasser atlas resolution. Each element in the parcellated adjacency matrix is computed as the mean of the links between the original nodes. Each connectome is an aggregation over 50 subject-specific connectomes, each binarized at 1% density.

We used data from from 250 healthy young adults, aged 22–35 years, from the human connectome project (HCP), which offers high-resolution in vivo whole-brain dMRI and resting-state fMRI (Van Essen et al., 2013). Structural and FC are derived from dMRI and fMRI, respectively. In dMRI, white matter trajectories across cortex are tracked probabilistically and used to form structural connectomes. In fMRI, the time-varying neural activity in each voxel is indirectly measured through the BOLD response and the Pearson correlation coefficient used to establish region-to-region FC graphs (Biswal, Yetkin, Haughton, & Hyde, 1995). Omitting subcortical and cerebellar information, the data contained 59,412 nodes covering the neocortex after removing the medial wall. The 250 subject functional and structural graphs were binarized by thresholding each graph at 1% density (keeping only the strongest links). Then, the 250 subjects were split into five nonoverlapping groups of 50 graphs. For each group, a single functional and structural graph was created by summing the individual graphs, resulting in five structural and five functional integer-weighted graphs with values *a*_*i*,*j*,*s*_ ∈ {1, …, 50}. Due to the high dimensionality, we resort to visualizing example graphs in the Glasser atlas (Glasser et al., 2016), which contains 360 areas (180 areas per hemisphere) (Figure 3).

## RESULTS

### Synthetic Data

Table 1 illustrates the effect of the mixing parameter *α* on the NMI between the expected partition **z**_*exp*_ and the maximum a posteriori (MAP) partition **z**_*MAP*_, and thereby indicates how robust the MSBM is at detecting population differences. When *α* is gradually increased, the difference between the two population cluster link probability matrices, *η*_*p*1_ and *η*_*p*2_, decreases, becoming identical for *α* = 1. As a result, the model becomes less robust. This implies that a certain level of population differences is required in order to be detectable by the MSBM, an effect that increases for higher number of clusters *K*. Assuming an acceptable median NMI of approximately 0.8 and above, the detection threshold appeared at *α* = 0.6 for *K* = 2, *α* = 0.5 for *K* = 5, and *α* = 0.2 for *K* = 10 for the balanced setting and at *α* = 0.7 for *K* = 2, *α* = 0.4 for *K* = 5, and *α* = 0.3 for *K* = 10 for the unbalanced setting. We consider the difference in these detection thresholds fairly small across the two settings. The model therefore appears to be relatively robust to cluster size heterogeneity.

**Table 1.**
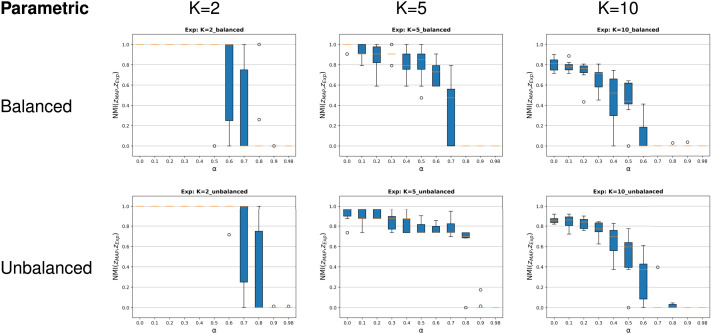
NMI, a measure of information overlap, for synthetic experiments with parametric MSBM. NMI was computed between the expected partition **z**_*exp*_ and the learned MAP partition **z**_*MAP*_ over 10 random initializations as a function of the mixing parameter *α* = {0, 0.1, … 0.98} (see Figure 2) for varying parameter combinations of cluster sizes “Nc_type” (balanced or unbalanced) and original number of clusters *K* = {2, 5, 10}.

### HCP Data

**Figure 4.**
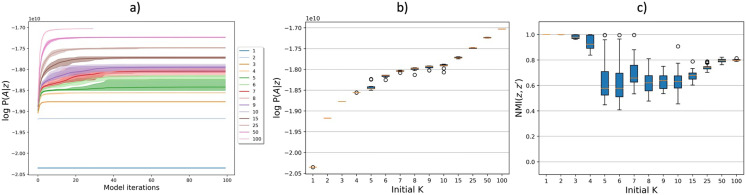
MSBM convergence diagnostics across the different number of initial clusters, *K* on HCP structural and functional connectomes. (A) Log likelihood as function of model iteration. The legend indicates *K*. (B) Highest likelihood sample log *P*($\bar{A}$∣**z**). (C) Pairwise NMI for different MAP partitions across 10 random initializations. The box extends from the first quartile (Q1) to the third quartile (Q3) of the data and the line marks the the median. The error bars (whiskers) extend from the box to the farthest data point lying within 1.5× the interquartile range from the box. Samples beyond this point (outliers) are represented by circles. The largest model of *K* = 100 was for computational reasons only, run for 30 iterations. For (B) and (C), “Initial K” refers to the number of clusters, which may be reduced by the model during inference.

In order to inspect model convergence, assess optimal number of clusters (according to the marginalized joint distribution Equation 4), and model robustness for different number of clusters, we conducted experiments for varying initial number of clusters *K* = {1, 2, …, 10, 15, 25, 50,100} across 10 random initializations. Since the joint distribution *P*(**z**, $\bar{A}$) and likelihood *P*($\bar{A}$∣**z**) are almost identical, we resort to only visualizing the likelihood. Figure 4A shows the log likelihood convergence across Gibbs iterations for the different *K* values. Here, we observed that the log likelihood was higher for higher *K*. This behavior was expected due to the increased model complexity. Furthermore, for *K* = {6, 7, …, 10}, the convergence across the random initializations was less stable around Gibbs iterations 10–40, but stabilized after the 40th Gibbs iteration. Figure 4B provides the highest obtained log likelihood *P*($\bar{A}$∣**z**) across different initial numbers of clusters *K*, whereas Figure 4C shows the pairwise NMI of the MAP partition obtained over multiple random initializations, that is, {NMI(**z**_1_, **z**_2_), NMI(**z**_2_, **z**_3_), …, NMI(**z**_10_, **z**_1_)}. In general, the NMI was high: For runs with *K* = {1, 2, 3, 4, 50, 100}, the NMI was 0.8 or above, which indicates that the model is robust and reproduces similar MAP partitions for these values of *K*. For *K* = {5, 6, …, 10, 15, 25}, the NMI was below 0.8, which indicates that the model is less robust. However, the NMI for *K* = 25 was close to 0.8 and can, thus, be viewed as the transition between the stable and semistable regions. We further observed that the gain of using more than 25 clusters was not substantial compared to the relative increase in model complexity.

**Figure 5.**
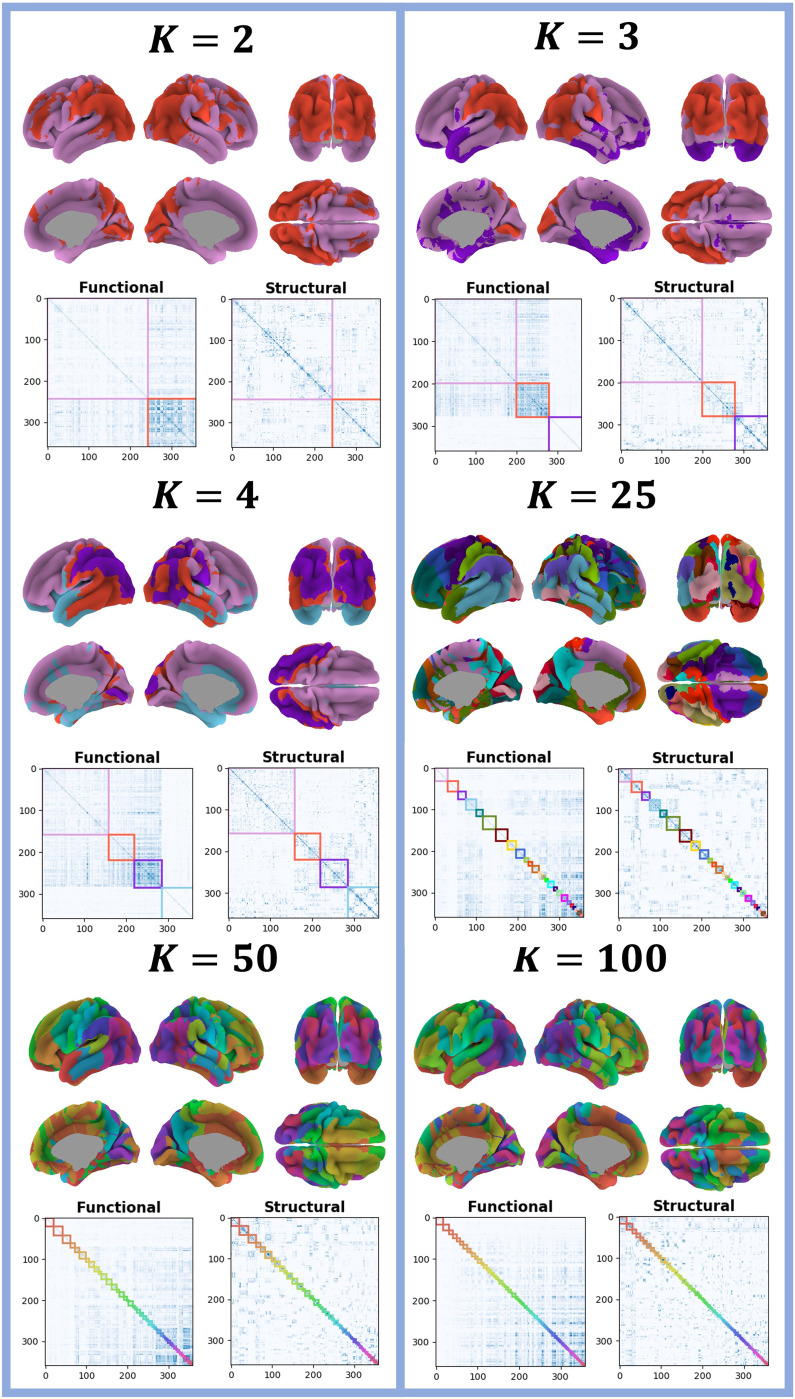
Brain maps for the learned partitions as well as adjacency matrices in Glasser resolution sorted according to the partitions for initial *K* = {2, 3, 4, 25, 50, 100}. Partitions in adjacency matrices were sorted according to their size (the color bar indicates the average link strength).

The learned partitions (brain maps) for *K* = {2, 3, 4, 25, 50, 100} are shown in Figure 5, along with the adjacency matrices in Glasser resolution sorted wrt. the learned partitions. The resulting brain maps were all spatially homogeneous, that is, contiguous nodes predominantly clustered together. Furthermore, there appears to be a hierarchical structure in the overall clustering pattern, that is, the pink cluster, containing frontotemporal areas in the brain map for *K* = 2 that was subdivided into an anterior temporal (purple) and a frontal-posterior temporal cluster (pink) for *K* = 3, while the red cluster (containing occipital and parietal areas) was somewhat preserved. This behavior was also observed when going from *K* = 3 to *K* = 4. Another notable observation is that all clusters for *K* = {2, 3, 4} were bihemispheric, whereas for *K* = {25, 50, 100}, some clusters were unihemispheric. However, these clusters appeared to have a “twin”-cluster on the opposite hemisphere. From the sorted adjacency matrices, the extracted regions displayed clear connectivity differences across the functional and structural graphs. For *K* = 2, the largest and smallest cluster had few and many functional links, respectively. For *K* = {3, 4}, the occipitoparietal cluster red had many functional links, whereas the anterior temporal cluster purple had few functional links. Notably, we observed a clear lack of FC in the anterior temporal lobe cluster for *K* = 3 (purple), *K* = 4 (purple), and *K* = 25 (orange, cluster 2), which we will explore further below.

**Figure 6.**
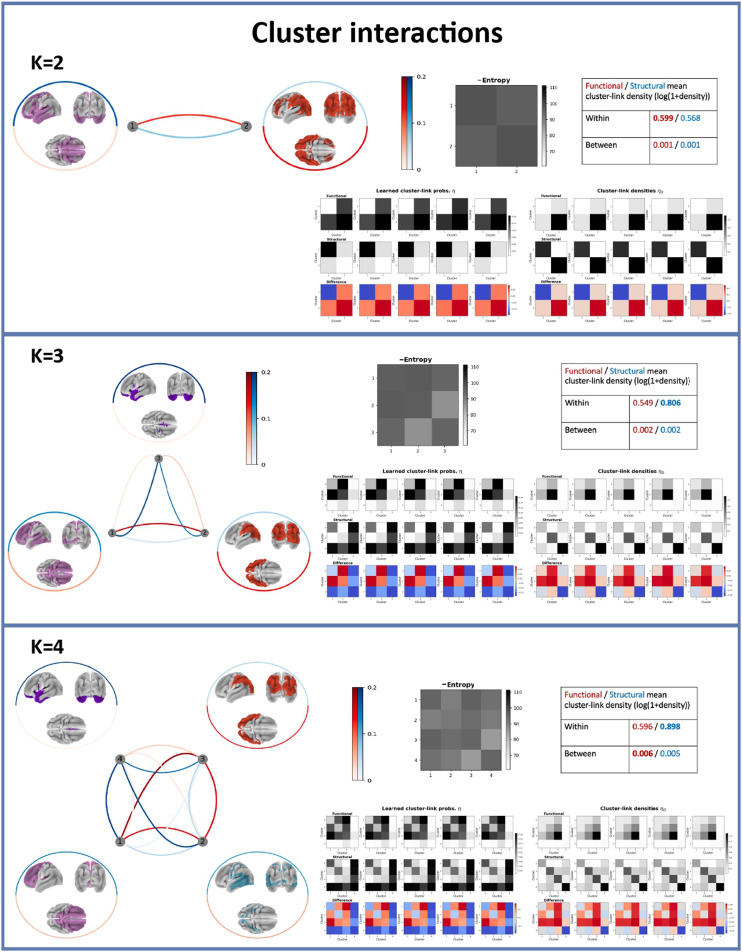
Cluster interactions for *K* = {2, 3, 4} illustrated by mean cluster link probabilities across functional (red) and structural (blue) connectomes, negative entropy matrix, individual (learned) cluster link probability matrices and cluster link density matrices (prior to modeling), as well as the differences between the respective functional and structural matrices. The line color and width in the graph indicate the link strength and negative entropy, respectively. The intercluster links are shown as lines between the respective clusters, whereas the intracluster links are shown as circles around the respective cluster.

Figure 6 shows cluster interactions from the learned link probability matrices $\bar{\eta }$ based on SC and FC differences for each of *K* = {2, 3, 4}. For each solution, we display the extracted segregated regions and highlight with red and blue colors the associated mean cluster link probabilities for the functional and structural connectomes, respectively. Furthermore, we show the original link densities (i.e., prior to modeling) within each cluster as well as structure-function differences for each inferred link. Notably, neither link probabilities nor link densities differed across the population. Thus, the model is driven by across-modality connectivity differences, and a low cluster link probability for one modality will lead to a higher cluster link probability for the other. Therefore, we also provide the (negative) entropy of the cluster link probabilities across the multiple graphs (see Equation 9) to assess the importance of the differences.

For *K* = 2, according to the entropy matrix, all links were almost equally important. The strongest (and slightly more dominant) links were intracluster links: For the frontotemporal cluster, the structural within-cluster link probability was strongest, whereas for the occipitoparietal (red) cluster, the functional intracluster link probability was strongest. However, the link density (prior to modeling) showed equal intracluster structural link density in the two clusters. Thus, discrepancies in intracluster FC in occipitoparietal and frontotemporal regions were the most prominent differences. Next, we observed higher between-cluster functional than structural link probability.

For *K* = 3, according to the entropy matrix, all links were again almost equally important; however, cluster pair red–purple (occipitoparietal vs. anterior temporal) was less noteworthy, explained by the low link density (i.e., prior to modeling) in both the functional and structural graphs for this cluster pair. The strongest link probabilities observed were the functional one between cluster pair pink–red (frontal-posterior temporal vs. occipitoparietal) and the structural one between cluster pair pink–purple and within the anterior temporal lobe cluster. This points to a prominent difference being that the frontal and posterior temporal lobe are more functionally connected to the parietal and occipital lobes, but both clusters are more structurally connected to the anterior temporal lobe. Also, the anterior temporal lobe showed low within- and between-cluster functional link probability, fortified by the aforementioned low functional link density in this region.

For *K* = 4, we observed that the anterior temporal lobe (purple in *K* = 3) and occipitoparietal (red in *K* = 3) clusters were unchanged, whereas the pink cluster from *K* = 3 (frontal lobe and posterior part of temporal lobe) was subdivided into a frontal lobe cluster (pink) and a posterior temporal lobe cluster (cyan). Here, we consistently observed higher functional intercluster connectivity between Clusters 1, 2, and 3, whereas the anterior temporal lobe cluster (Cluster 4) was substantially more structurally than functionally connected to itself and the other extracted clusters. Considering the intracluster connectivity, Clusters 1, 2, and 4 are more structurally than functionally connected to themselves, whereas Cluster 3 is more functionally connected to itself.

For *K* = 3 and *K* = 4, we also observed a higher within-cluster SC than FC, suggesting that structural connections are more constrained to the learned clusters, whereas FC is more widespread.

**Figure 7.**
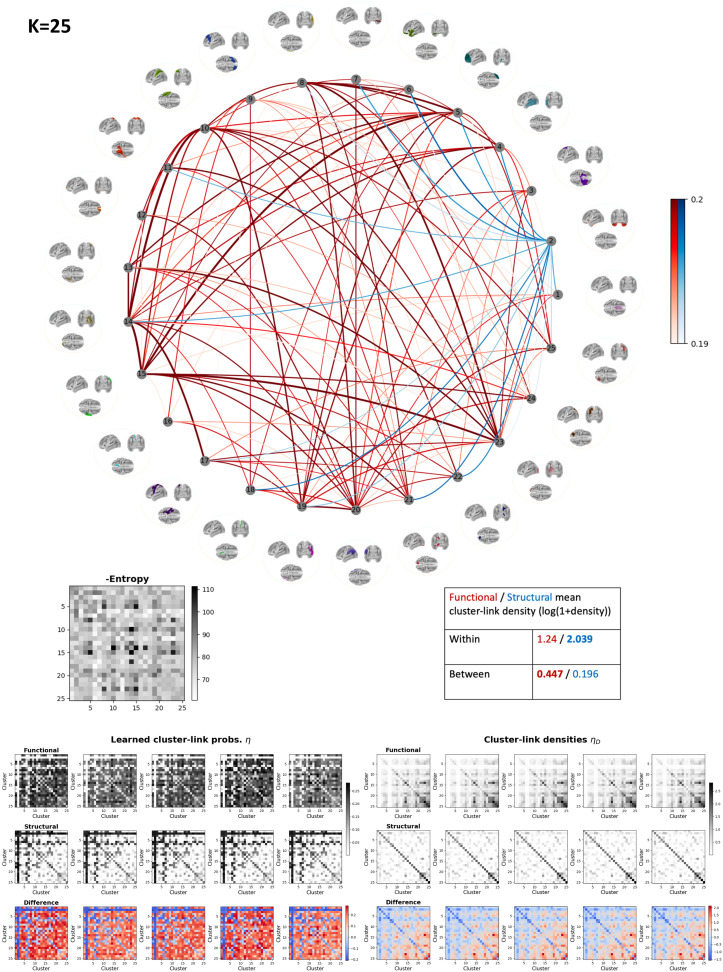
Cluster interactions for *K* = 25 illustrated by the top 20% mean cluster link probabilities across functional (red) and structural (blue) connectomes, negative entropy matrix, and individual (learned) cluster link probability matrices and cluster link density matrices (prior to modeling), as well as the differences between the respective functional and structural matrices. The color bar has been adjusted to the respective range (80–100th percentile) to allow for easier visualization of prominent links.

In Figure 7, we display the connectivity pattern for the top 20% link probabilities for the *K* = 25 model. The functional intercluster links were the most prominent links (strongest and highest negative entropy). According to the entropy matrix, the most important links were between the cluster pairs 5–8, 5–15, 8–23, 10–14, 14–15, 14–17, and 15–23, which are all functional links. This highlights that the integration between Clusters 5 (left-frontal gyrus), 8 (right parietal gyrus), 10 (left primary somatosensory cortex), 14 (right middle occipital lobe), 15 (right primary auditory cortex), 17 (left primary motor cortex), and 23 (left middle occipital lobe) are important when differentiating SC and FC. The SC was observed to be more restricted. This also aligns with the finding in Figure 6, where the structural link density is generally higher *within* each cluster and spatially close structures are more structurally connected. As such, we observe from the mean within- and between-cluster link densities that SC is mostly manifested in terms of within-cluster links, while FC is comparatively more prominent in terms of between-cluster links. The only prominent structural links were links between the bilateral anterior temporal lobes (Cluster 2) and most of the other clusters. However, when considering the cluster link densities, it should be noticed that this cluster has near-zero functional link density, resulting in higher learned structural cluster link probability. From the difference in link densities, we further observe that Clusters 6 and 7 also show higher structural between-cluster connectivity. Notably, these clusters also included anterior temporal regions.

The clear separation of the anterior temporal lobe from other clusters in having very low FC may be attributed to the fMRI data suffering from signal loss in this region. The included connectomes were originally computed for a previous publication dating back some years (Albers et al., 2021); we recomputed the connectomes using updated preprocessing pipelines (Supporting Information Figure S1), as well as sorted by the inferred partitions from current models (Supporting Information Figure S2). For this purpose, the most recent Independent Component Analysis (ICA)-fix denoised and preprocessed fMRI data were downloaded from HCP, and structural connectomes were computed according to the recent state-of-the-art tractography methods (Mansour L., Di Biase, Smith, Zalesky, & Seguin, 2023), including gyral bias reduction. Individual connectomes were here binarized at 0.1% density instead of the previous 1%. However, we still clearly noticed a loss of functional signal in the anterior temporal lobes as opposed to strong structural links. Thus, even if these results may be attributed to problems with the data acquisition methods, these have not been solved by recent advances in preprocessing.

To further illuminate the reason for the significance of the anterior temporal lobe, where FC is near zero, we computed the power spectral densities of the fMRI data for each node and subject, and averaged over nodes within each cluster and across subjects (see Supporting Information Figure S3). While the data were highpass-filtered at 2,000 s as part of the HCP denoising pipeline (Smith et al., 2013), the anterior temporal lobe cluster (purple for *K* = 3 and *K* = 4, Cluster 2 for *K* = 25) clearly had the most high-frequency content. When normalizing the spectra for each subject and cluster, these spectra for these areas were near flat, suggesting that the information in the frequency band typically associated with BOLD fMRI, approximately 0.01 to 0.1 Hz is relatively low in this region.

To provide an additional assessment of the inferred clusters, we counted the within- and between-cluster structural and functional connectome links of 125 participants not included in the 250 subjects used to learn clustering partitions. Followingly, we produced paired *t* tests for each cluster–cluster link count, and visualized the *p* value of significant links (see Supporting Information Figure S4). Many links were highly significant, with some *p* values on the order of 10^−120^. Most links were significant at Bonferroni-corrected significance level, *α* = 0.001/(*K* · (*K* + 1)/2). Of these, the anterior temporal lobe cluster showed the strongest statistical significance. These results suggest that the inferred clusters generalize to unseen subjects.

To further highlight the difference between the inferred partitions and existing parcellations, we computed the adjusted mutual information (Vinh, Epps, & Bailey, 2010) between several atlases in the HCP space defined at different levels of resolution (Arslan et al., 2018) (see Supporting Information Figure S5). Evidently, the inferred MSBM partitions differ from existing parcellations, which is expected since the MSBM optimizes for differences across the pool of graphs, in this case, SC and FC.

## DISCUSSION

In this article, we investigated prominent differences between structural and functional human brain connectivity using a novel approach featuring an MSBM that infers graph vertices systematically differing across a set of adjacency matrices. The structural and functional connectomes were derived from dMRI and fMRI, respectively, from 250 HCP subjects. The 250 binary adjacency matrices were collapsed into five structural and five functional integer-weighted group-level connectomes by summing the connectivity of 50 nonoverlapping subjects. Being a parametric model, the MSBM allows defining the scale at which the segregated structures should be inferred, that is, the number of clusters.

We produced MSBM solutions for a range of number of clusters *K* (see Figure 4) and found, based on similarity between random initializations of the same models, high clustering stability for *K* = {2, 3, 4, 50, 100} (NMI ≥ 0.8). For *K* = {5, …, 10, 15}, we found that 0.6 < NMI < 0.8; thus, we consider the model for these *K* values as semistable (somewhat more stable than unstable). *K* = 25 was considered a transition point for the model, since the NMI starts increasing again. It should be noted that high clustering stability does not necessarily imply an optimal partition, but rather that the model consistently produced similar partitions across random initializations. The small increase in data likelihood and NMI by increasing model complexity from *K* = 25 to *K* = 50 was not deemed sufficient to visualize cluster interactions for the model with larger *K* from a perspective of maintaining parsimony. The resulting brain maps, shown in Figure 5, were spatially homogeneous and split hierarchically upon increasing *K*. We further found that for low resolution maps (*K* = {2, 3, 4}), all clusters were bihemispheric with prominent differences represented by comparatively low FC for the anterior temporal lobe and stronger FC within and between the other extracted clusters. When increasing the number of clusters (*K* = {25, 50, 100}), we also observed unihemispheric clusters, and for the *K* = 25 solution, it was evident that intercluster connections were functionally stronger than structurally, including interhemispheric connections. The anterior temporal lobe was an exception and was characterized by the absence of functional connections across scale, a finding further validated by having highly statistically significant structure-function differences on unseen data. The comparatively stronger intercluster FC, including interhemispheric ones, we attribute to SC having challenges tracking through the corpus callosum (Crawford, 2022; Jeurissen et al., 2019; Jones, 2010; Škoch et al., 2022).

We further explored the weak functional connections in the anterior temporal lobe by examining the power spectra of the fMRI data. While other clusters consistently showed a peak in the frequency band most commonly associated with BOLD fMRI (0.01–0.1 Hz), the anterior temporal lobe spectrum was much flatter. This indicates a high presence of high-frequency noise in the fMRI data for this area, and consequently, functional correlations with other areas can be assumed to be influenced by this noise and approaching zero. One possible explanation is that the highlighted area is at the bottom of the brain, which perhaps were not coil-covered sufficiently. Nevertheless, these results indicate a spatial bias in the HCP fMRI data consistently present across subjects and preprocessing pipelines, which results in temporally noisy data specifically for the anterior temporal lobe. Additionally, these results underscore the importance of knowing and investigating limitations of the data when analyzing MSBM results.

Importantly, both structural and functional connectomes are derived from imperfect imaging techniques. Structural connectomes are built from tractography on diffusion-weighted imaging, which suffers from a bias toward short-range fibers, gyral bias (Schilling et al., 2018), that is, the predominance of tracking streamline endpoints at gyri rather than sulci even though cortical thickness is invariant, and a probabilistic estimation framework dependent on a preselected number of streamlines (Schilling et al., 2018). Tractography streamline methods generally suffer from an inability to accurately identify crossing fibers and has a predominance toward intrahemispheric connections as also observed. Similarly, functional connectomes were computed as time averages, even though brain connectivity is well known to be time varying (Chang & Glover, 2010; Olsen et al., 2022).

We also conducted preliminary experiments (not shown) with a nonparametric version of the MSBM (with a Chinese restaurant process prior on *K*; not shown), which enables the model to select the optimal number of clusters from an initial value (Mørup et al., 2014). In general, the nonparametric version would be preferable to the parametric one, since it is fully data driven, and results would not depend on predefined model parameters. However, we found that the inferred number of clusters exploded when trained on real data, which corresponds well with the fractal nature of high-resolution neuroimaging data (Varley et al., 2020). Instead, it may be more advantageous to search for brain networks of a size that more easily provides meaningful interpretation. By applying a parametric model instead, we explicitly control the scale at which prominent differences should be derived, by specifying a maximum number of clusters that cannot be exceeded during inference. Thus, when applied to neuroimaging data, the parametric version of the model is preferable to the nonparametric version. However, future avenues should aim to develop alternative sampling methods for the parametric model, allowing a more efficient search of the parameter space, such as the split-merge sampler used for the nonparametric model, which may be better in high dimensions or when the posterior distribution has a multimodal shape (Mørup et al., 2014).

The MSBM can be applied to various network resolutions and we here demonstrated that it is flexible enough to handle the HCP network in full resolution (approximately 60,000 nodes). Whether processing higher or lower resolution data is necessary is very problem dependent. Notably, our high-resolution analyses showcase that it is possible to process and obtain detailed maps specifically identifying regions that optimally differ across graphs for a user-specified number of clusters. The MSBM therefore poses as a useful tool to uncover (pinpoint and describe) connectivity differences. Furthermore, when using atlas-defined low-resolution representations, details can be missed and areas collapsed that would otherwise be differentiable.

Our synthetic experiment results in Table 1 suggest that the MSBM requires a certain level of population differences in order to detect them. However, given graphs with significant population differences, the model is robust in detecting these differences. For the synthetic connectomes, we found that the MSBM’s ability to detect the differences became less robust for a higher number of clusters. Specifically, the detection threshold for *K* = {2, 5, 10} was at *α* = {0.6, 0.4, 0.2}, respectively, implying that higher resolution maps of differences demands that the data exhibit more prominent population differences. Furthermore, varying the cluster sizes, as opposed to having identical cluster sizes, did not appear to affect the MSBM performance. It should, however, be noted that this property was only tested for predefined unbalanced cluster sizes, where the total number of nodes was 100 and the largest difference in cluster sizes was for *K* = 5 with a minimum of five nodes and maximum of 60 nodes per cluster. In the balanced setting, we, in general, expect the detection threshold to occur simultaneously for clusters having the same size and levels of connectivity differences, whereas for the unbalanced setting, we expect that small clusters will fail earlier but larger clusters will be more robust which enables their identification at lower levels of differences due to their larger size and thus larger statistical support.

By using a multinomial likelihood in the MSBM, we allow modeling of both binary and integer-weighted graphs. As such, the model directly generalizes to integer-weighted graphs similar to how SBMs have been generalized to integer-weighted graphs by use of the Poisson distribution (see also Peixoto, 2019; Schmidt & Morup, 2013). However, the model does not admit modeling of continuous or negative weights as, for instance, implemented for SBM by use of a Gaussian likelihood (Aicher, Jacobs, & Clauset, 2015; Herlau, Mørup, Schmidt, & Hansen, 2012). We exploit that the MSBM handles integer-weighted graphs to collapse the 250 binary subject graphs into five integer-weighted structural and functional graphs, respectively. This aggregation disables the opportunity for subject-specific post hoc investigations. We note that the computational burden when modeling high-resolution graphs (∼60,000 nodes) is significant (approximately 24-hr central processing unit (CPU) time for 100 Gibbs iterations for *K* = 4). Notably, to scale the MSBM to larger graphs, more scalable inference procedures should be considered, such as inference scaled by graphics processing unit (GPU) acceleration and stochastic variational inference procedures (Hoffman, Blei, Wang, & Paisley, 2013). While not featured here, subject-specific post hoc investigation is entirely possible, since the MSBM produces graph-specific ***η*** cluster link probability matrices.

The aim of this study was to establish that the MSBM can be used to characterize discrepancies across connectomes as demonstrated in terms of characterising the prominent differences in structural and function connectomes quantified from fMRI/dMRI. We therefore presently considered differences across groups of subjects, while the framework could easily be used for discovering temporal differences in graphs derived from a single subject, such as task-related connectivity changes or effect of medication or the analysis of connectivity differences within a modality across a larger cohort of subjects. Within-subject studies would probably be less affected by data acquisition biases than in the present study. In particular, the MSBM can be used in general to identify optimally differentiating network structures across a series of graphs and can thus be employed within a modality to characterize the variability across laboratories, subjects, health and disease, or tasks, rather than modality-specific differences as presently considered. Consequently, the MSBM approach can have important future applications in general within neuroscience studies aiming to characterize changes in connectivity across connectomes.

### Conclusion

This article provides a valuable methodology to efficiently uncover prominent differences across a set of graphs. By explicitly characterizing the *differences* between resting-state FC and SC using a generalized formulation of the SBM, that is, the MSBM, we obtained detailed maps optimally describing differentiating properties of SC and FC in terms of segregated clusters that systematically differ in connectivity patterns across the two modalities. Synthetic experiments highlighted the MSBM’s robustness in detecting population differences, albeit with decreased robustness for higher number of clusters, resulting in less statistical support and thus more prominent population differences are required. When applied on the HCP data, the MSBM again demonstrated robustness for varying number of clusters and across random initializations with a NMI ≥ 0.8. The inferred brain maps were spatially homogeneous and consistent over all clustering patterns when varying the number of clusters. For low-resolution brain maps (*K* = {2, 3, 4}), the most prominent differences were observed for bihemispheric clusters primarily pointing to low FC for the bilateral anterior temporal lobes, whereas for the higher resolution brain map (*K* = 25), some prominent differences were observed for unihemispheric clusters as well. Our work showcases how MSBM can offer valuable insights in terms of mapping the prominent structural and functional brain connectivity differences. However, we anticipate that the MSBM has a general use where differences across multiple connectomes are of interest.

## ACKNOWLEDGMENTS

This projected was supported by Ingeborg and Leo Dannins scholarship for scientific research as well as Independent Research Fund Denmark (Grant Number 0136-00315B) granted to Morten Mørup.

**WU-Minn HCP Consortium Open Access Data**

Data were provided [in part] by the Human Connectome Project, WU-Minn Consortium (Principal Investigators: David Van Essen and Kamil Ugurbil; 1U54MH091657) funded by the 16 NIH Institutes and Centers that support the NIH Blueprint for Neuroscience Research and by the McDonnell Center for Systems Neuroscience at Washington University.

## SUPPORTING INFORMATION

Supporting information for this article is available at https://doi.org/10.1162/netn_a_00399.

## AUTHOR CONTRIBUTIONS

Nina Braad Iskov: Conceptualization; Formal analysis; Investigation; Methodology; Software; Validation; Visualization; Writing – original draft; Writing – review & editing. Anders Stevnhoved Olsen: Conceptualization; Data curation; Formal analysis; Investigation; Methodology; Validation; Visualization; Writing – original draft; Writing – review & editing. Kristoffer Hougaard Madsen: Conceptualization; Data curation; Investigation; Methodology; Supervision; Writing – original draft; Writing – review & editing. Morten Mørup: Conceptualization; Data curation; Funding acquisition; Investigation; Methodology; Project administration; Resources; Supervision; Writing – original draft; Writing – review & editing.

## FUNDING INFORMATION

Morten Mørup, Ingeborg and Leo Dannins scholarship for scientific research. Morten Mørup, Danmarks Frie Forskningsfond (https://dx.doi.org/10.13039/501100011958), Award ID: 0136-00315B.

## Supplementary Material



## TECHNICAL TERMS

Connectome: A graph adjacency matrix, where rows and columns typically represent brain areas, and matrix entries represent their anatomical or functional interconnection.
Graph: A conceptual representation of relationships between nodes/vertices connected by links/edges (terms used interchangeably). Can be directed or undirected, binary or weighted, etc.
Stochastic block model (SBM): An unsupervised Bayesian model that partitions nodes of a graph into clusters based on the adjacency matrix link density within and between clusters.
Multinomial SBM (MSBM): An unsupervised Bayesian model that partitions nodes in a set of graphs based on maximally varying within/between cluster link density across the set of graphs.
Partition: A division of nodes into nonoverlapping, clusters/groups.
Adjacency matrix: A specific way of representing the graph using a matrix that may be manipulated using typical mathematical operations.
Gibbs sampling: Approximate inference method used for inferring parameters facing intractable model evidence that requires the evaluation of all possible parameter settings.
Parsimony: Also known as Occam’s razor, parsimony describes the principle to search for the simplest solution without sacrificing performance.
