# Supplementary material for: Discovering prominent differences in structural and functional connectomes using a multinomial stochastic block model
Source: Netw Neurosci. 2024 Dec 10;8(4):1243–64. doi: 10.1162/netn_a_00399 (PMC11674489; doi:10.1162/netn_a_00399)
Supplement: Supplementary file 1 [file netn-8-4-1243-s001.pdf]

Journal: NETWORK NEUROSCIENCE

METHODS

Supplementary Material: Discovering Prominent Differences in Structural and Functional Connectomes Using a Multinomial Stochastic Block Model

Nina Braad Iskov[1], Anders Stevnhoved Olsen[1], Kristoffer Hougaard Madsen[1,2] and Morten Mørup[1]

[1] Department of Applied Mathematics and Computer Science, Technical University of Denmark, Lyngby, Denmark

[2] Danish Research Centre for Magnetic Resonance, Centre for Functional and Diagnostic Imaging and Research, Copenhagen University Hospital - Amager and Hvidovre, Copenhagen, Denmark

Keywords: [Structural connectivity, Functional connectivity, Multinomial Stochastic Block Model, Difference modeling, Bayesian inference]

SUPPLEMENTARY MATERIALS

Code

Code is available here: github.com/Ninaiskov/ConnDiff-MultSBM.

Model distribution derivations

The MSBM uses a Multinomial likelihood for $\overline{\eta}$, which is discrete and thus allows modelling of both binary and integer-weighted graphs. A natural choice of prior on the link-probabilities is the Dirichlet distribution, due to its conjugacy. The prior distribution on the clustering for the parametric MSBM is

given by the Dirichlet-Multinomial distribution in Equation (1).

$$\begin{aligned} p(\boldsymbol{z}) &= \int p(\boldsymbol{z}, \boldsymbol{\pi}) d\boldsymbol{\pi} \\ &= \frac{\Gamma(K\alpha)}{\Gamma(K\alpha + N)} \prod_{k=1}^{K} \frac{\Gamma(n_k + \alpha)}{\Gamma(\alpha)} \\ &= \frac{\Gamma(K\alpha)}{\Gamma(K\alpha + N)\Gamma(\alpha)^K} \prod_{k=1}^{K} \Gamma(n_k + \alpha) \\ &= \frac{B(\mathbf{n} + \boldsymbol{\alpha})}{B(\boldsymbol{\alpha})}, \end{aligned} \tag{1}$$

which can be reparameterized using $\alpha := \alpha/K$ such that we obtain

$$\begin{aligned} p(\mathbf{z}) &= \frac{\Gamma(\alpha)}{\Gamma(\alpha + N)\,\Gamma(\alpha/K)^K} \prod_{k=1}^{K} \Gamma(n_k + \alpha/K) \\ &= \frac{B(\mathbf{n} + \alpha/K \cdot \mathbf{1})}{B(\alpha/K \cdot \mathbf{1})}. \end{aligned} \tag{2}$$

To complete the model formulation, we need to specify the Dirichlet prior on the cluster-link probabilities as well as the Multinomial likelihood of node-links. The **Dirichlet** prior on the link probability between cluster l and m ("cluster-link probability") across all graphs S is given by

$$p\left(\overline{\boldsymbol{\eta}}_{l,m} \middle| \overline{\boldsymbol{\eta}}_0\right) = \mathrm{Dir}\left(\overline{\boldsymbol{\eta}}_0\right) = \frac{1}{B(\overline{\boldsymbol{\eta}}_0)} \prod_{s=1}^{S} \eta_{l,m,s}^{\eta_{0,s}-1}, \tag{3}$$

where $\overline{\boldsymbol{\eta}}_{l,m}$ denotes the cluster-link probability vector across graphs for cluster pair l, m. $\boldsymbol{\eta}_0$ denotes the concentration parameter vector across graphs. The Beta function $B(\cdot)$ and Gamma function $\Gamma(\cdot)$ are related by

$$\frac{1}{B\left(\overline{\boldsymbol{\eta}}_0\right)} = \frac{\Gamma\left(\sum_{s=1}^{S} \eta_{0,s}\right)}{\prod_{s=1}^{S} \Gamma\left(\eta_{0,s}\right)}. \tag{4}$$

The **Multinomial** likelihood of the link between node i and j across all graphs S is given by

$$p\left(\overline{\mathbf{a}}_{i,j} | \overline{\boldsymbol{\eta}}_{z_i,z_j}\right) = \frac{\Gamma\left(\sum_{s=1}^{S} a_{i,j,s} + 1\right)}{\prod_{s=1}^{S} \Gamma\left(a_{i,j,s} + 1\right)} \prod_{s=1}^{S} \eta_{z_i,z_j,s}^{a_{i,j,s}}, \tag{5}$$

where $\overline{\mathbf{a}}_{i,j}$ denotes the link vector across graphs for node pair i, j.

Marginalized likelihood derivation

In order to obtain $p\left(\overline{\mathbf{A}}|\boldsymbol{z}\right)$ we need to marginalize out the nuisance parameter $\boldsymbol{\eta}$, i.e.

$$p\left(\overline{\mathbf{A}}|\boldsymbol{z}\right) = \int p\left(\overline{\mathbf{A}}|\overline{\boldsymbol{\eta}}_{z_i,z_j}\right) p\left(\overline{\boldsymbol{\eta}}\right) d\boldsymbol{\eta}. \tag{6}$$

Using definitions of the Multinomial likelihood and Dirichlet prior, given in Equation (5) and Equation (3) we get

$$\begin{aligned} p\left(\overline{\mathbf{A}}|\boldsymbol{z}\right) &= \int \underbrace{\prod_{i>j} \frac{\Gamma\left(\sum_{s=1}^{S} a_{i,j,s}+1\right)}{\prod_{s=1}^{S}\Gamma\left(a_{i,j,s}+1\right)}}_{\text{const. wrt. } \eta} \prod_{s=1}^{S} \eta_{(z_i,z_j),s}^{a_{i,j,s}} \prod_{l\geq m} \frac{1}{B(\overline{\boldsymbol{\eta}}_0)} \prod_{s=1}^{S} \eta_{l,m,s}^{\eta_{0,s}-1} d\boldsymbol{\eta} \\ &= C \int \prod_{i\geq j} \prod_{s=1}^{S} \eta_{(z_i,z_j),s}^{a_{i,j,s}} \prod_{l\geq m} \frac{1}{B(\overline{\boldsymbol{\eta}}_0)} \prod_{s=1}^{S} \eta_{l,m,s}^{\eta_{0,s}-1} d\boldsymbol{\eta}, \end{aligned} \tag{7}$$

where C denotes the terms constant wrt. $\boldsymbol{\eta}$. Using the relation $(z_i, z_j) \in (l, m)$ and the product rule for exponents, we can sum the exponents of $\eta_{l,m,s}$

$$p\left(\overline{\mathbf{A}}|\boldsymbol{z}\right) = C \int \prod_{l\geq m} \frac{1}{B\left(\overline{\boldsymbol{\eta}}_0\right)} \prod_{s=1}^{S} \eta_{l,m,s}^{\sum_{z_i\in l, z_j\in m} a_{i,j,s}+\eta_{0,s}-1} d\boldsymbol{\eta}. \tag{8}$$

We now define the $\nu_{l,m,s} \equiv \sum_{z_i\in l, z_j\in m} a_{i,j,s}$ as the number of links between cluster pair l, m for each graph s. Using this notation, the vector containing the number of links between cluster pair l, m distributed across graphs will be denoted $\overline{\boldsymbol{\nu}}_{z_i,z_j} = \overline{\boldsymbol{\nu}}_{l,m}$. We can now recognize the purple term from the Dirichlet distribution with concentration parameter $\overline{\boldsymbol{\nu}}_{l,m} + \overline{\boldsymbol{\eta}}_0$, i.e.

$$\mathrm{Dir}\left(\overline{\boldsymbol{\nu}}_{l,m} + \overline{\boldsymbol{\eta}}_0\right) = \frac{1}{B\left(\overline{\boldsymbol{\nu}}_{l,m} + \overline{\boldsymbol{\eta}}_0\right)} \prod_{s=1}^{S} \eta_{l,m,s}^{\nu_{l,m,s}+\eta_{0,s}+1}. \tag{9}$$

Since probability distributions must integrate to one, we know that

$$\int \prod_{s=1}^{S} \eta_{l,m,s}^{\nu_{l,m,s}+\eta_{0,s}+1} d\boldsymbol{\eta} = B\left(\overline{\boldsymbol{\nu}}_{l,m} + \overline{\boldsymbol{\eta}}_0\right), \tag{10}$$

such that

$$\frac{B\left(\overline{\boldsymbol{\nu}}_{l,m} + \overline{\boldsymbol{\eta}}_0\right)}{B\left(\overline{\boldsymbol{\nu}}_{l,m} + \overline{\boldsymbol{\eta}}_0\right)} = 1. \tag{11}$$

Using the result in Equation (10) and changing index back to l, m, we can reduce Equation (8) to

$$p\left(\overline{\mathbf{A}}|\boldsymbol{z}\right) = C \prod_{l\geq m} \frac{B\left(\overline{\boldsymbol{\nu}}_{l,m} + \overline{\boldsymbol{\eta}}_0\right)}{B\left(\overline{\boldsymbol{\eta}}_0\right)}. \tag{12}$$

When comparing the marginalized likelihood in Equation (12) with Equation (1), we see that we end up with the Dirichlet-Multinomial distribution as expected.

Parametric Gibbs sampler: Conditional posterior derivation

In order to derive the Gibbs sampler, or more specifically: the conditional posterior of node i belonging to a new cluster d, we start by applying Bayes' rule to expand the expression

$$\underbrace{p\left(z_i = d|\overline{\mathbf{A}}, \boldsymbol{z}^{\setminus i}\right)}_{\text{Conditional posterior}} = \frac{p\left(\overline{\mathbf{A}}, z_i = d|\boldsymbol{z}^{\setminus i}\right)}{\sum_{d'} p\left(\overline{\mathbf{A}}, z_i = d'|\boldsymbol{z}^{\setminus i}\right)}, \tag{13}$$

where the conditional joint probability is given by

$$p\left(\overline{\mathbf{A}}, z_i = d|\boldsymbol{z}^{\setminus i}\right) = \underbrace{p\left(\overline{\mathbf{A}}|z_i = d, \boldsymbol{z}^{\setminus i}\right)}_{\text{Conditional likelihood}} \underbrace{p\left(z_i = d|\boldsymbol{z}^{\setminus i}\right)}_{\text{Conditional prior}}. \tag{14}$$

We will start with deriving the **conditional likelihood**. We know that the full likelihood $p\left(\overline{\mathbf{A}}|\boldsymbol{z}\right)$ is defined in Equation (12). Since the constant terms does not influence the Gibbs sampler, we only consider the terms that changes wrt. the cluster assignment, i.e.

$$p(\overline{\mathbf{A}}|\boldsymbol{z}) \propto \prod_{l \geq m} B\left(\overline{\boldsymbol{\nu}}_{l,m} + \overline{\boldsymbol{\eta}}_0\right). \tag{15}$$

Note in Equation (12) that besides the constant C, $\frac{1}{B(\boldsymbol{\eta}_0)}$ is also constant over all clusters. In order to derive the conditional likelihood $p\left(\overline{\mathbf{A}}|z_i = d, \boldsymbol{z}^{\setminus i}\right)$, we perform the following steps:

1. remove the contribution of z_i (cluster assignment for node i),
2. remove contribution of cluster d
3. add the contribution of assigning node i to the cluster d

Following the steps above, we get

$$\begin{aligned} p\left(\overline{\mathbf{A}}|z_i = d, \boldsymbol{z}^{\setminus i}\right) &= \frac{\prod_m^K B\left(\overline{\boldsymbol{\nu}}_{d,m} + \overline{\boldsymbol{\eta}}_0\right)}{\prod_m^K B\left(\overline{\boldsymbol{\nu}}_{d,m}^{\setminus i} + \overline{\boldsymbol{\eta}}_0\right)} \prod_{l \geq m} B\left(\overline{\boldsymbol{\nu}}_{l,m}^{\setminus i} + \overline{\boldsymbol{\eta}}_0\right) \\ &\propto \frac{\prod_m^K B\left(\overline{\boldsymbol{\nu}}_{d,m} + \overline{\boldsymbol{\eta}}_0\right)}{\prod_m^K B\left(\overline{\boldsymbol{\nu}}_{d,m}^{\setminus i} + \overline{\boldsymbol{\eta}}_0\right)}. \end{aligned} \tag{16}$$

Next we derive the **conditional prior**: We know that the full parametric prior $p(\boldsymbol{z})$ is defined by Equation (1). As before, we only consider the terms that changes wrt. the cluster assignment, i.e.

$$p(\boldsymbol{z}) \propto \prod_{k=1}^{K} \Gamma\left(n_k + \alpha\right) \tag{17}$$

In order to get $p(z_i = d|\boldsymbol{z}^{\setminus i})$, we perform the same steps as before, i.e.

$$\begin{aligned} p\left(z_i = d|\boldsymbol{z}^{\setminus i}\right) &= \frac{\Gamma(n_d + \alpha)}{\Gamma\left(n_d^{\setminus i} + \alpha\right)} \prod_{k=1}^{K} \Gamma\left(n_k^{\setminus i} + \alpha\right) \\ &\propto n_d^{\setminus i} + \alpha. \end{aligned} \tag{18}$$

Note that the blue term is constant for each cluster assignment d, thus it does not influence the Gibbs sampler. The final expression $n_d^{\setminus i} + \alpha$ is the result of having Gamma functions in both numerator and denominator. Substituting Equation (16) and Equation (18) into Equation (14) yields the conditional joint probability:

$$p\left(\overline{\mathbf{A}}, z_i = d|\boldsymbol{z}^{\setminus i}\right) \propto \frac{\prod_m^K B\left(\overline{\boldsymbol{\nu}}_{d,m} + \overline{\boldsymbol{\eta}}_0\right)}{\prod_m^K B\left(\overline{\boldsymbol{\nu}}_{d,m}^{\setminus i} + \overline{\boldsymbol{\eta}}_0\right)} (n_d^{\setminus i} + \alpha). \tag{19}$$

Synthetic data

Table 1. Unbalanced cluster sizes. Since N is 100, each Nc list must sum to 100.

K	Nc list (number of nodes per cluster)
2	[70, 30]
5	[60, 20, 10, 5, 5]
10	[20, 20, 10, 10, 10, 10, 5, 5, 5, 5]

Table 2. Synthetic initial partitions z_{ini} used for generating synthetic adjacency matrices. Each partition indicate the node assignment of N=100 nodes into $K = [2, 5, 10]$ clusters. The node distribution across the clusters is either balanced or unbalanced. Note that the initial partition will not necessarily be the same as the partition expected to be found by the MSBM. The expected partition will be a partition showing the "difference"-clusters across the population.

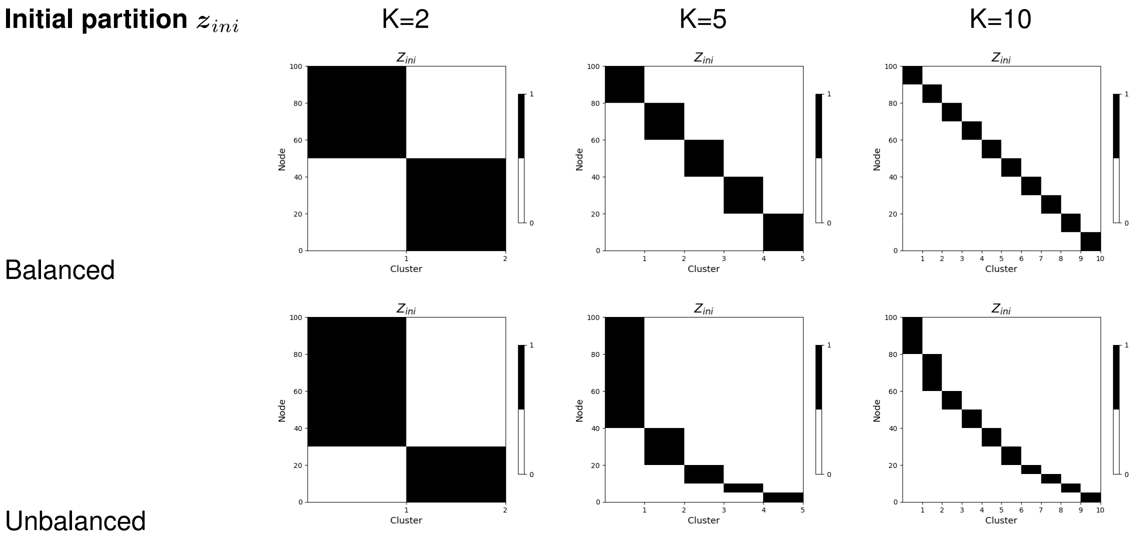

Table 3. Synthetic cluster-link probability matrices for different original number of clusters K and similarity scales α. Notice that for $\alpha = 0.5$, the difference between the cluster-link probs. is less prominent than for $\alpha = 0$.

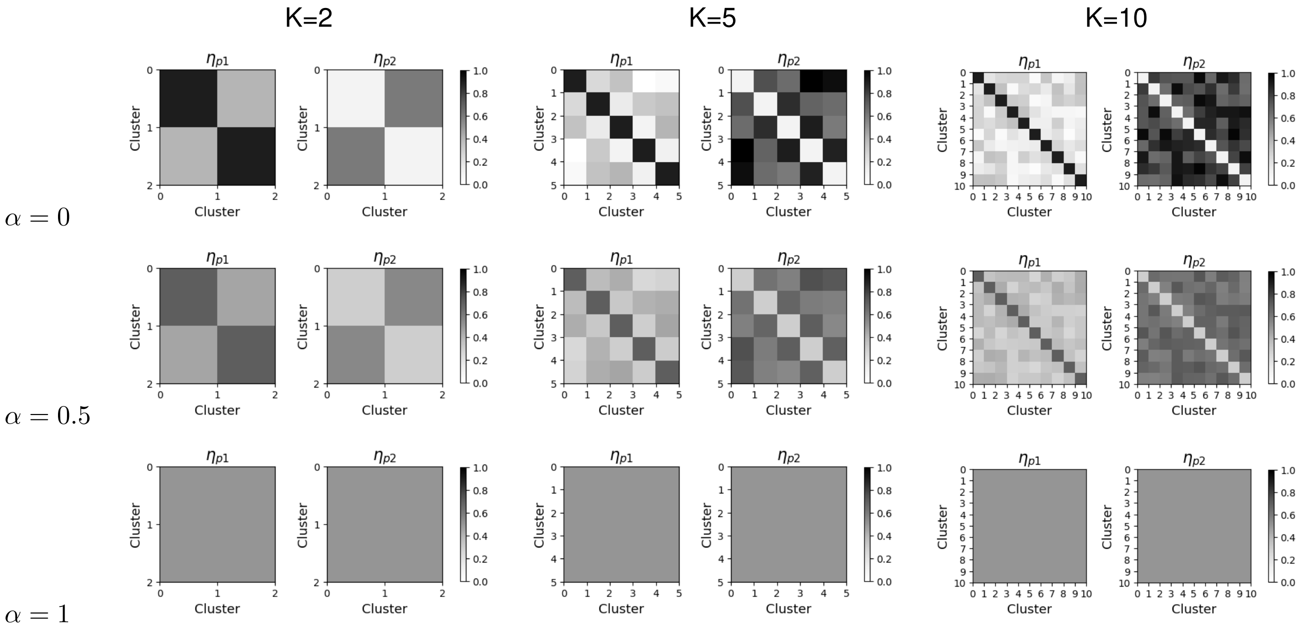

Table 4. Expected partition matrices $\boldsymbol{z}_{exp}$. Example for Nc_type=unbalanced. Notice that for $0 < \alpha < 1$, $\boldsymbol{z}_{exp} = \boldsymbol{z}_{ini}$. This is because no matter how close to 0 α is, η_{p1} and η_{p2} will be elementwise different, thus all original clusters are "difference"-clusters.

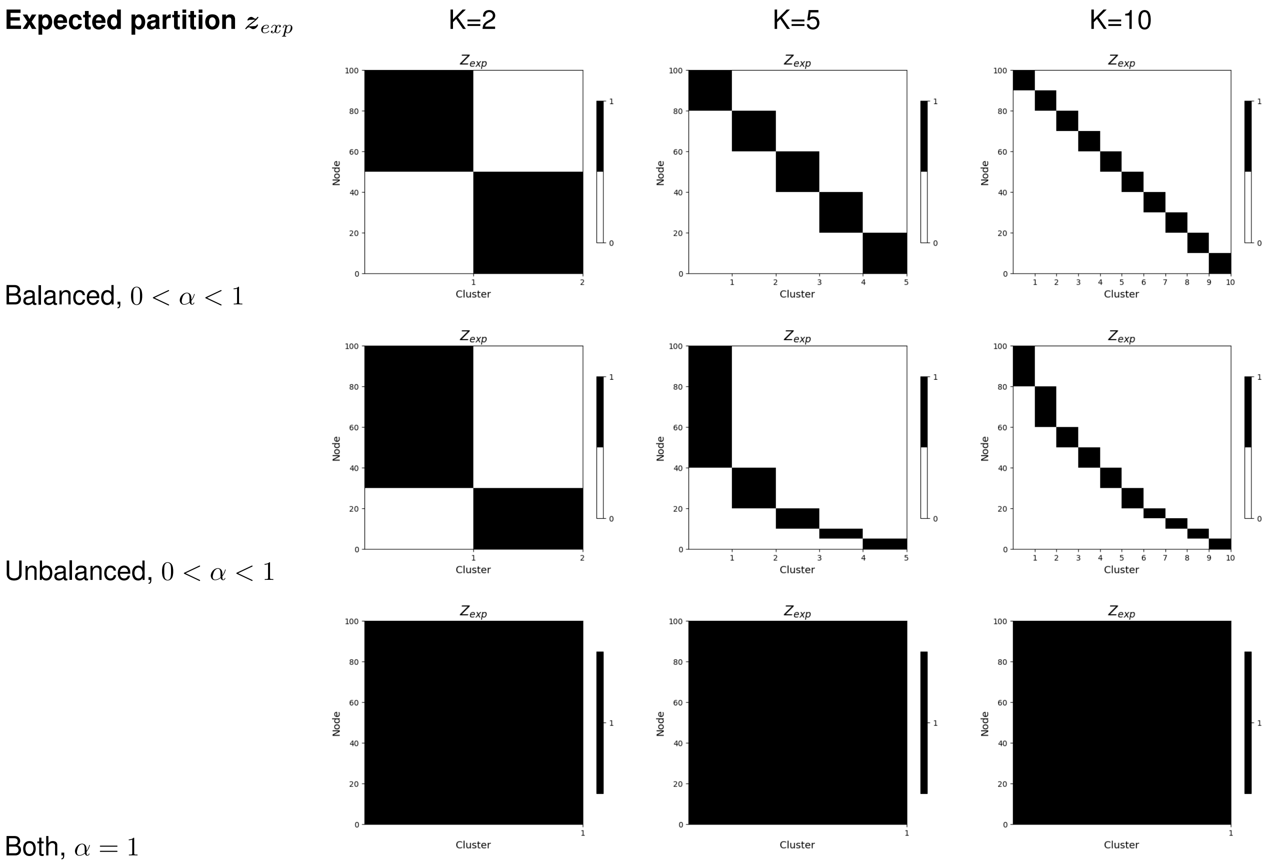

HCP data: extra results

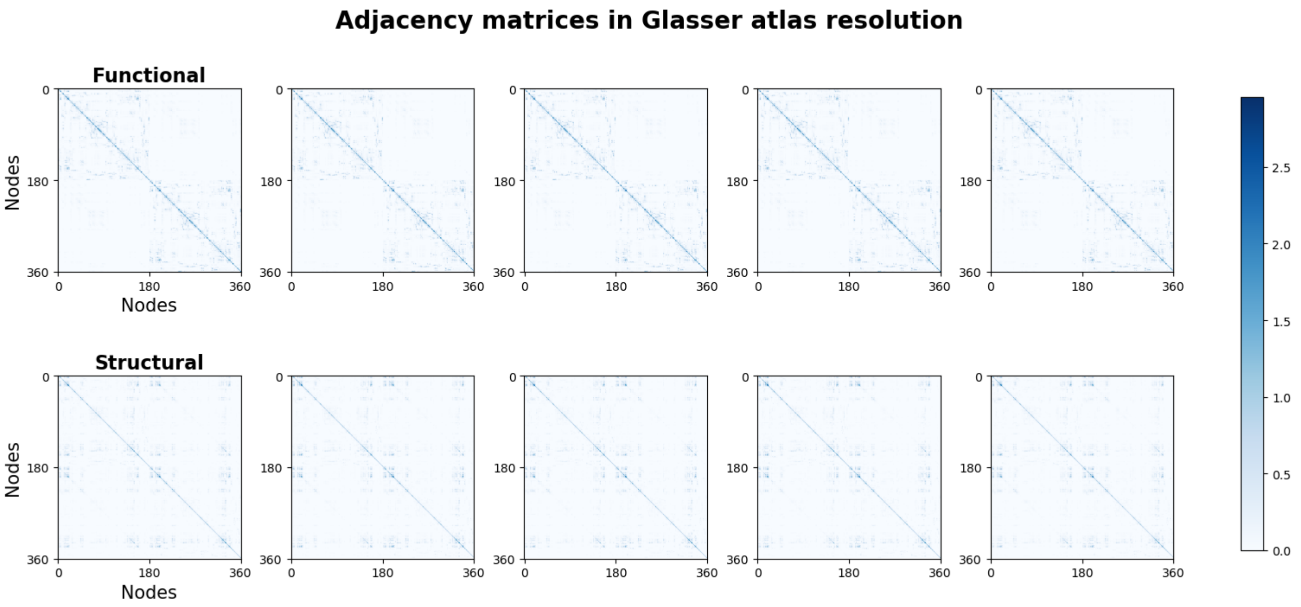

Figure 1. Graphs obtained using most recent HCP preprocessing pipeline and visualized in Glasser atlas resolution. Each element in the parcellated adjacency matrix is computed as the mean of the links between the original nodes.

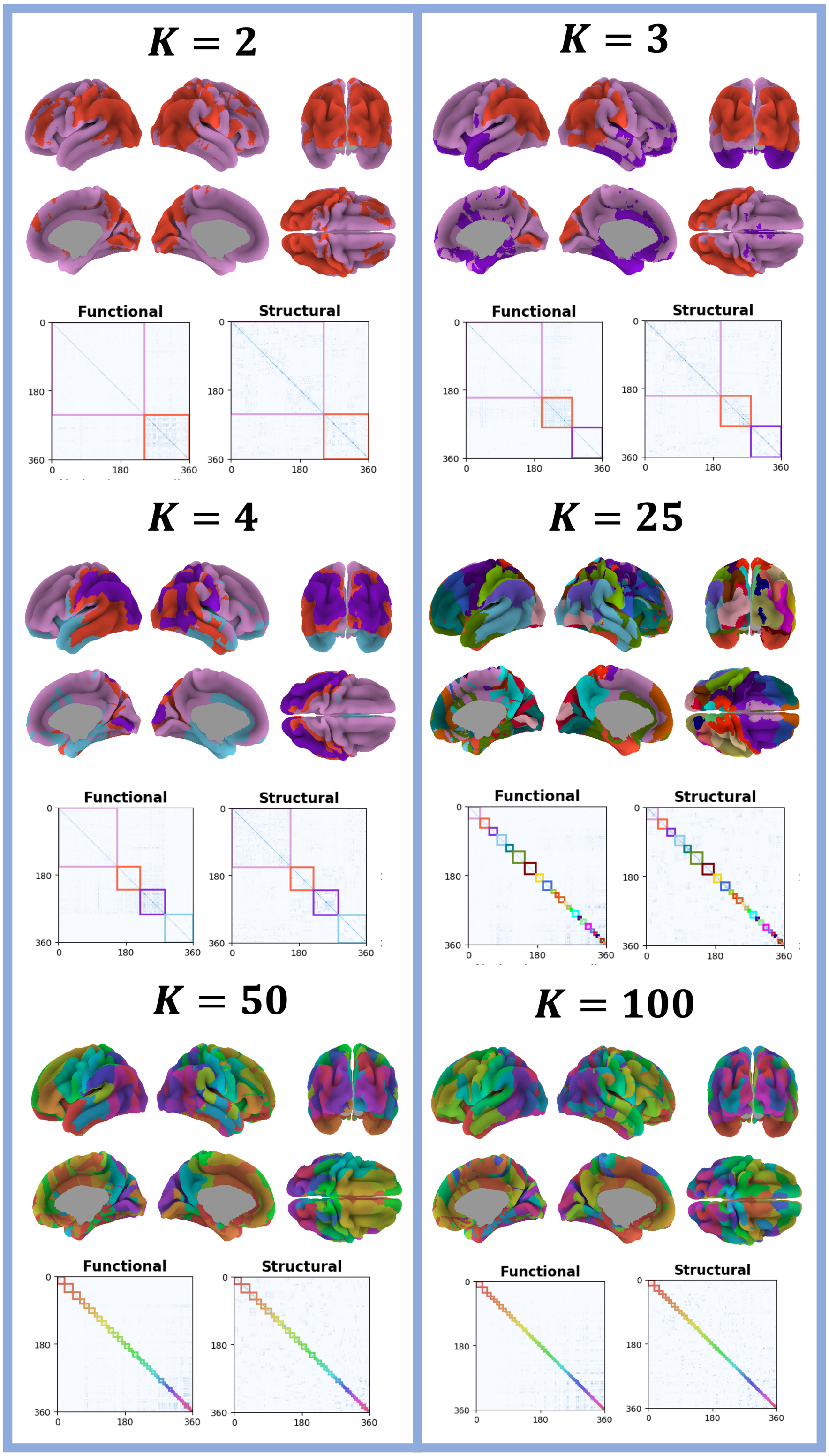

76 **Figure 2.** Brain maps for the learned partitions as well as adjacency matrices obtained using most recent HCP preproccessing pipeline visualized in Glasser
77 resolution sorted according to the partitions for initial $K = \{2, 3, 4, 25, 50, 100\}$. Partitions in adjacency matrices were sorted according to their size, and the
78 colorbar indicates average link strength.

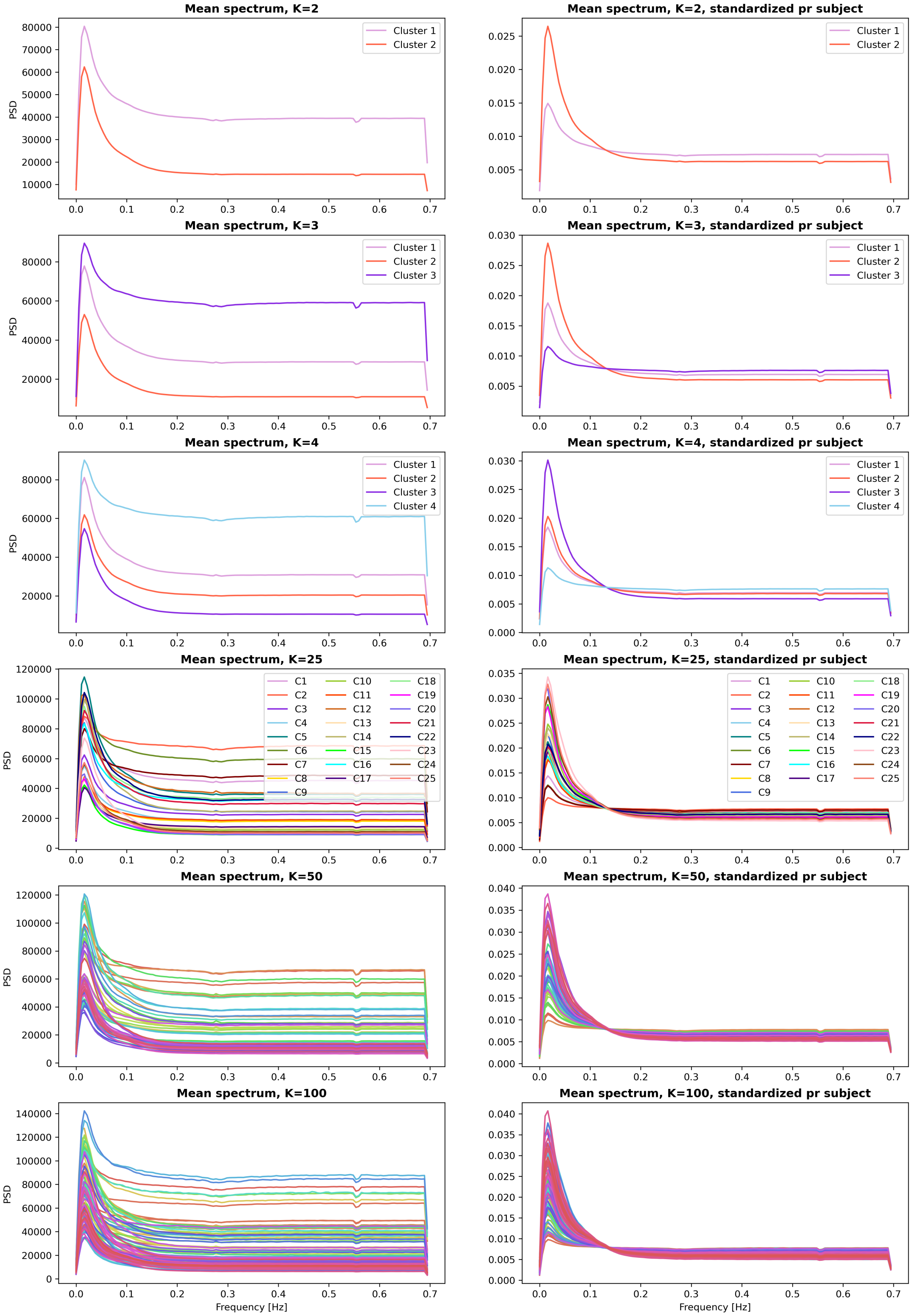

Figure 3. Average fMRI spectrum across nodes assigned to each cluster using inferred partitions. For the plots to the right, each spectrum was divided by its sum to display the power distribution over frequencies. Note that certain clusters (e.g., C3 for K=3, C4 for K=4, and C2 for K=25) display a more flat normalized power spectrum which we attribute to an increased noise level in the fMRI data, subsequently leading to reduced meaningful correlations with other clusters.

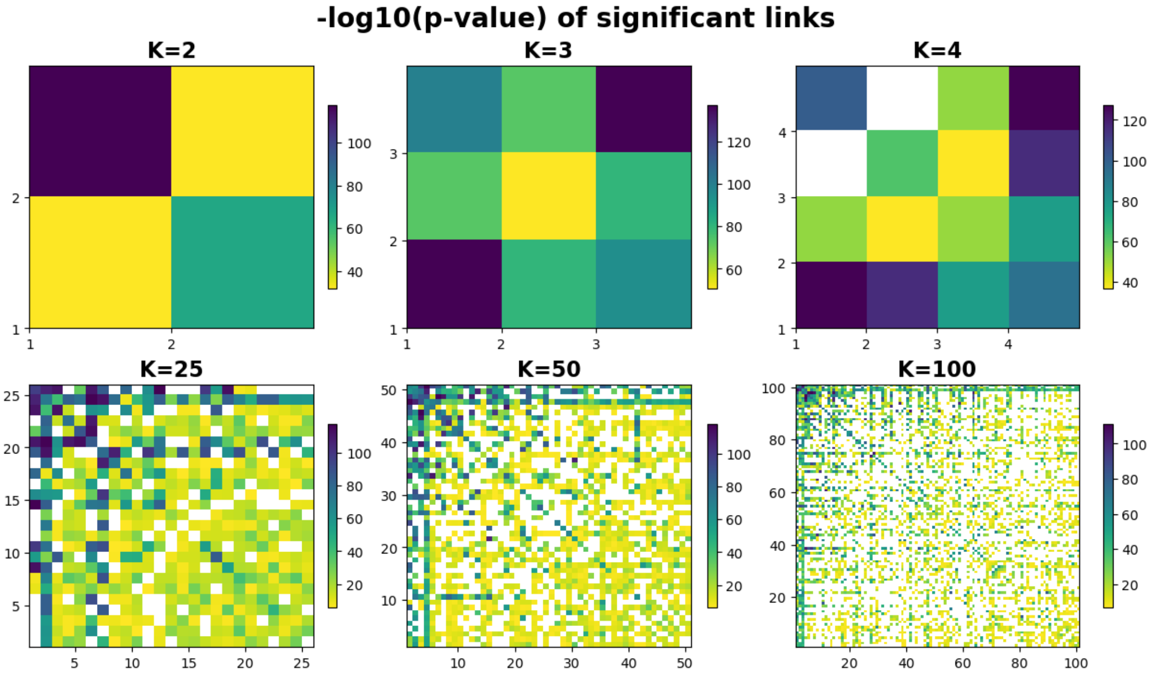

Figure 4. P-values of significant links according to statistical test (paired t-test) on cluster-link densities "etaD" (tested on unseen data: 125 new HCP subjects with significance level $\alpha = 0.001$ which is Bonferroni corrected, such that $\alpha_{bonf} = \alpha/(K \times (K-1)/2 + K)$). All colored elements represent significant links, whereas the white elements are insignificant links. Since most elements are significant, the test shows that the difference between functional and structural inter-cluster link density are indeed significant. Note that low values indicate low p-value, i.e. more significant (bright/yellow color)

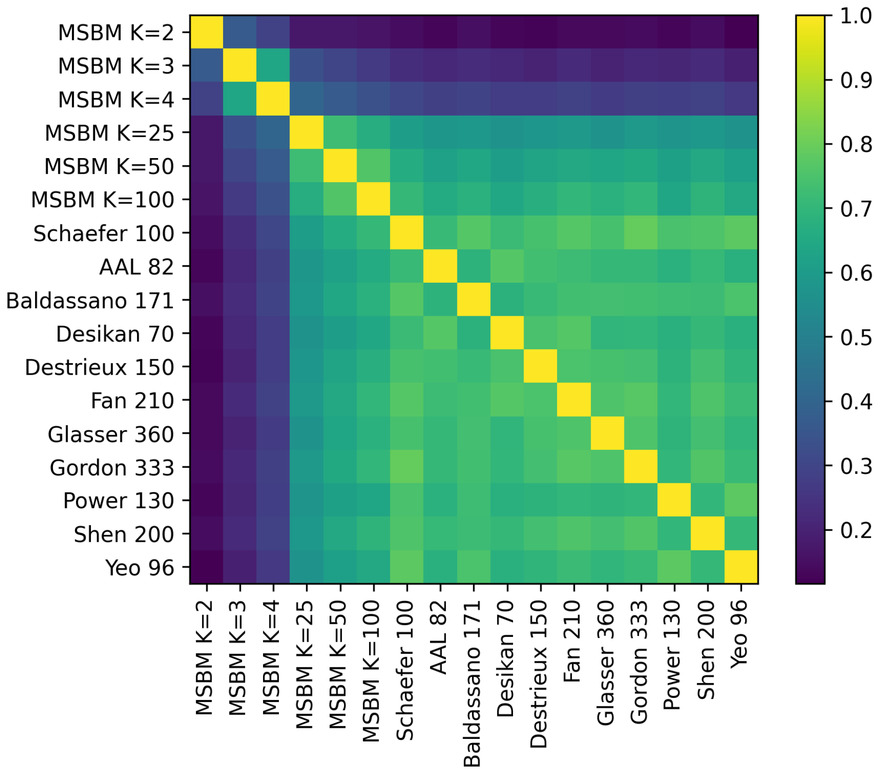

87 **Figure 5.** Adjusted mutual information between our clustering solutions and several known parcellations.
